# Clinical characteristics and outcomes of paediatric acute lymphoblastic leukaemia in a tertiary hospital in Tanzania: a single-centre observational study

**DOI:** 10.1186/s41182-025-00760-2

**Published:** 2025-05-27

**Authors:** Koki Shimizu, Koga Luhulla, Magreth Msoffe, Chambega Chambega, Salama Mahawi, Primus Ewald, Godlove Sandi, Irene Msirikale, Ruchius Philbert, Regina Kabona, Lulu Chirande, Nana Jacqueline Nakiddu, Patricia Scanlan, Chris Smith, Yasushi Miyazaki, Camille Maringe, Bernard Rachet, Hadija Mwamtemi

**Affiliations:** 1 School of Tropical Medicine and Global Health, Nagasaki University, 1-12-4 Sakamoto, Nagasaki, 852-8523 Japan; 2 Inequalities in Cancer Outcomes Network (ICON), Department of Health Services Research and Policy, Faculty of Public Health and Policy, London School of Hygiene and Tropical Medicine, 15-17 Tavistock Place, London, WC1H 9SH UK; 3 Department of Paediatrics and Child Health, Muhimbili National Hospital, Malik road, Dar es Salaam, P.O. Box 65000, Tanzania; 4 Department of Paediatrics and Child Health, Muhimbili University of Health and Allied Sciences, United Nations road, Dar Es Salaam, P.O. Box 65001, Tanzania; 5 Department of Haematology, Atomic Bomb Disease Institute, Nagasaki University, 1-12-4 Sakamoto, Nagasaki, 852-8523 Japan

**Keywords:** Acute lymphoblastic leukaemia, Sub-Saharan Africa, Paediatric, Global health, LMICs

## Abstract

**Background:**

A wide inequality exists between high- and low-income countries in the outcome of paediatric acute lymphoblastic leukaemia (ALL). At a tertiary-level hospital in Tanzania, multidimensional approaches have been taken to improve cancer care for children. This study aimed to update the outcomes of paediatric ALL at Muhimbili National Hospital (MNH), Tanzania from 2016 to 2020.

**Methods:**

We performed a retrospective chart review of children who were treated with modified UKALL2003 protocol at MNH from January 1, 2016 to December 31, 2020. We used the Cox proportional hazards model to estimate the effect of each prognostic factor on event-free survival (EFS).

**Results:**

We identified 202 patients who had confirmatory diagnoses of ALL and initiated treatment at MNH. Fifty-two patients (26%, 52/202) died (*n* = 47) or abandoned treatment (*n* = 5) before the end of remission induction. The main causes of death during this period were infections and bleeding complications. The median EFS was 9 months and 2-year EFS was 36%. Oedema, non-early rapid responder, and non-remission were associated with short EFS in the multivariable analysis.

**Conclusions:**

The number of new paediatric ALL admissions at MNH has doubled in the past decade. The prevention of early deaths is critical to improve the long-term survival of paediatric ALL in Tanzania.

**Supplementary Information:**

The online version contains supplementary material available at 10.1186/s41182-025-00760-2.

## Background

Acute lymphoblastic leukaemia (ALL) is the most common cancer among children, and accounts for one-third of the 400,000 children diagnosed with cancer every year worldwide [1, 2]. Paediatric ALL is highly curable in high-income countries (HICs) with a 5-year survival of over 90% [3]. However, a wide gap exists in its survival outcome between HICs and low- and middle-income countries (LMICs) [4]. ALL is listed as one of the six index cancers for the World Health Organization (WHO) Global Initiative for Childhood Cancer because of its high potential for cure [5].

One strategy to improve leukaemia outcomes in LMICs is to implement chemotherapy protocols that are adapted according to the local availability of resources [6]. The intensity of treatment and level of supportive care must be carefully balanced, so that patients do not die from the toxicity of treatment, but receive the optimal dose of treatment. The assessment of this balance should be performed regularly because the medical infrastructures in LMICs are constantly evolving [7].

In 2004, the Tanzanian government opened its first paediatric oncology ward of 17 beds in a national oncology facility, Ocean Road Cancer Institute. Patients who were treated on UKALL2003 protocol, majority without asparaginase, from 2008 to 2010 showed median event-free survival (EFS) of 12 months and 2-year EFS of 33% [8, 9]. Since then, the paediatric oncology service has made progress through the support of the government, the hospital, a local charity, and international partners. The paediatric oncology ward was incorporated into the paediatric department of a national tertiary hospital (Muhimbili National Hospital (MNH)) in 2012, and expanded its capacity to 65 beds with access to a paediatric intensive care unit. The service was further strengthened by developing training programs for paediatric oncology doctors and nurses, improving patient documentation, automating chemotherapy prescriptions, adapting the routine use of asparaginase products and intrathecal chemotherapy, increasing the availability of antibiotics and antifungals, gaining access to diagnostic tools (immunostaining and flow-cytometry) and measurable residual disease (MRD) measurement, building a local collaborative cancer network, and increasing the number and specialisation of ward staff including nutritionists and social workers.

Through these advancements in the local paediatric oncology program in the past two decades, this study aimed to update the clinical outcomes of paediatric ALL at MNH, Tanzania.

## Methods

### Setting

We performed a retrospective chart review of paediatric patients between 1 and 19 years of age who initiated treatment for ALL at the paediatric oncology ward of MNH in Dar es Salaam, Tanzania from January 1, 2016 to December 31, 2020. The data were collected as of June, 2022. The ward facilities and staff were summarised in Supplementary Table S1. Under the local paediatric cancer network, there were eight hospitals in Tanzania, seven of which provided only ALL maintenance treatment; and MNH, the largest paediatric oncology facility, that performed the whole course of ALL treatment from remission induction to maintenance.

We identified patients who initiated ALL treatment at MNH using hospital admission records; and their diagnoses were confirmed using patient records and the ALL database of the local paediatric cancer network. We collected information on patient demographics, clinical characteristics, and outcomes.

### Diagnoses

Diagnoses were confirmed based on the bone marrow or peripheral blood morphology at MNH, accompanied by flow cytometry of the appropriate diagnostic sample examined either at the MNH laboratory or at the laboratory of Children’s Health Ireland in Ireland. Diagnostic samples were sent to Ireland through the support of the local charity when the flow cytometry service was not available locally. Patients with mature B-cell ALL were excluded. However, due to limited access to cytogenetics, we were unable to accurately identify, and, therefore, exclude those with Philadelphia-chromosome-positive ALL.

### Chemotherapy

**Fig. 1:**
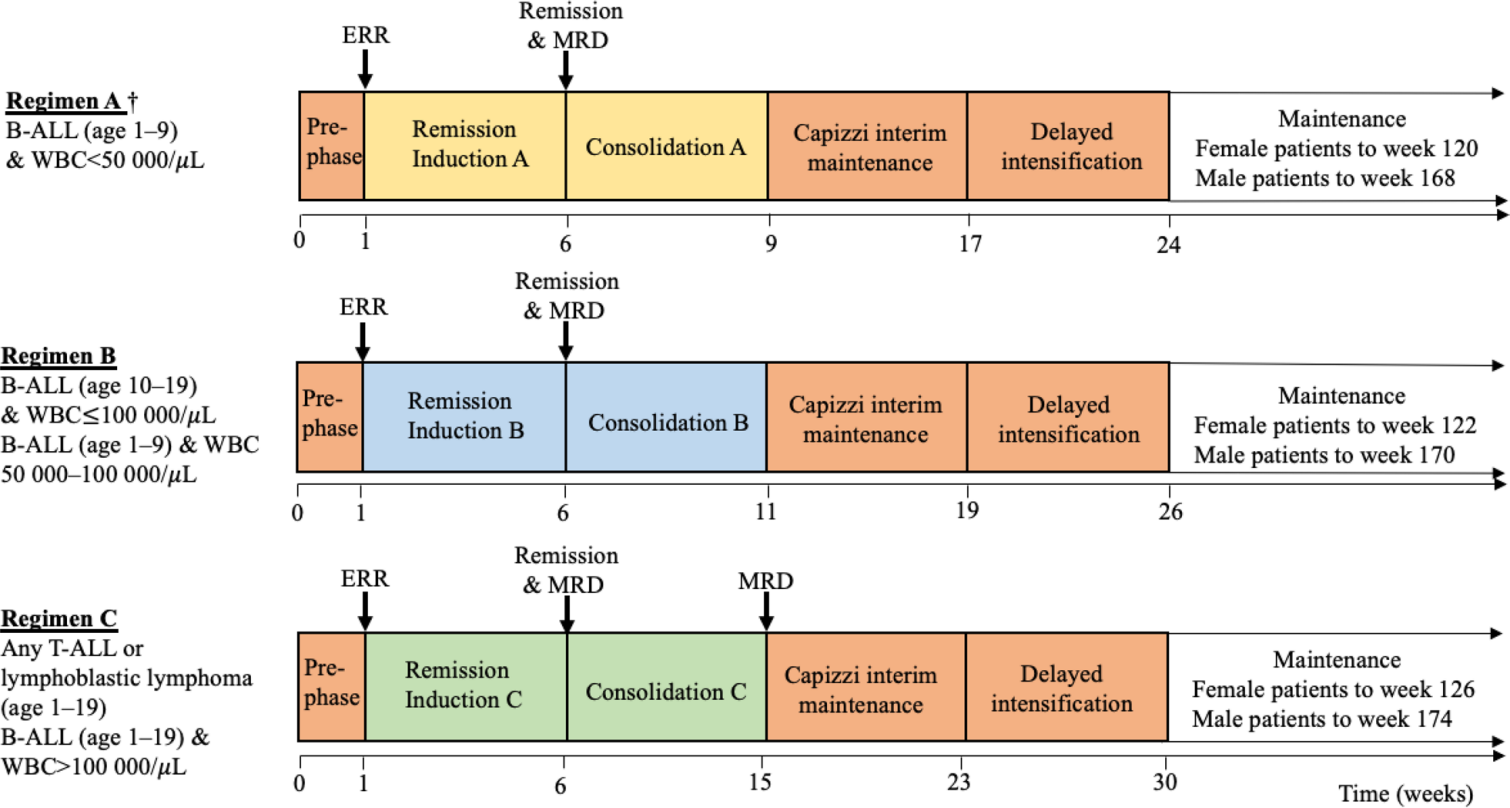
Outline of modified UKALL2003 protocol. This diagram describes the outline of modified UKALL2003 protocol. Patients were risk-stratified to receive one of three escalating-intensity regimens (**A**, **B**, **C**). The treatment content differs for remission induction and consolidation. All patients received one cycle of Capizzi interim maintenance and delayed intensification. ALL, acute lymphoblastic leukaemia; ERR, early rapid responder; MRD, measurable residual disease; WBC, white blood cell

Patients were treated with modified UKALL2003 protocol (Fig. 1, details in Supplementary Table S2). It differed from the original UKALL2003 protocol in that at MNH: (i) the protocol was preceded by a week of steroid treatment called pre-phase, (ii) L-asparaginase was used instead of pegylated asparaginase, (iii) all patients received the same treatment from Capizzi interim maintenance to maintenance regardless of the risk stratification, and (iv) they received only 1 cycle of Capizzi interim maintenance and delayed intensification.

Patients were initially assigned to one of three escalating-intensity regimens (A, B, or C) based on the type of ALL, patient age, and white blood cell (WBC) count at presentation. Regimen A was stopped in 2018 due to suspicion of high relapse, and those on regimen A were treated instead by regimen B.

Patients were assessed on their clinical response at three timepoints (Table 1); after pre-phase, after remission induction (RI), and after consolidation. Patients underwent early rapid responder (ERR) assessment after pre-phase. ERRs were defined as those who had clearance of blasts in peripheral smear taken on RI day 1, or less than 25% of blasts in the bone marrow taken on RI day 8. Patients who were non-ERRs and were on regimen A or B were shifted to regimen C. After RI, patients underwent remission and MRD assessment. Remission was defined as having less than 5% of blasts in the bone marrow, determined by morphology. MRD negativity was defined as having less than 0.1% of blasts in the bone marrow, determined by flow cytometry. If their treatment responses were inadequate, they were switched to a higher-intensity regimen arm or were palliated. Patients who received consolidation C underwent a second MRD assessment at the end of consolidation; if they were MRD positive, they were palliated.

**Table 1:** Assessment of clinical response in modified UKALL2003 protocol

ERR assessment (after pre-phase)	Response	ERR		Non-ERR	
	Clinical decision	Continue same regimen		Patients on regimen A or B, shift to C Patients on regimen C, continue C	
Remission and MRD assessment (after remission induction)	Response	BM blasts < 0.1%	BM blasts B: 0.1–5% T: 0.1–10%		BM blasts B: > 5% T: > 10%
	Clinical decision	Continue same regimen	Patients on regimen A or B, shift to C Patients on regimen C, continue C		Palliation
MRD assessment (after consolidation) * Applies only to those who received consolidation C	Response	BM blasts < 0.1%		BM blasts $\ge$ 0.1%	
	Clinical decision	Continue same regimen		Palliation	

BM, bone marrow; ERR, early rapid responder; Non-ERR, non-early rapid responder; MRD, measurable residual disease

### Clinical outcomes

The survival was assessed using EFS since the date of treatment initiation. Events were defined as death, relapse, being sent home for palliation, or abandonment of treatment, whichever occurred first.

### Statistical analysis

**Fig. 2:**
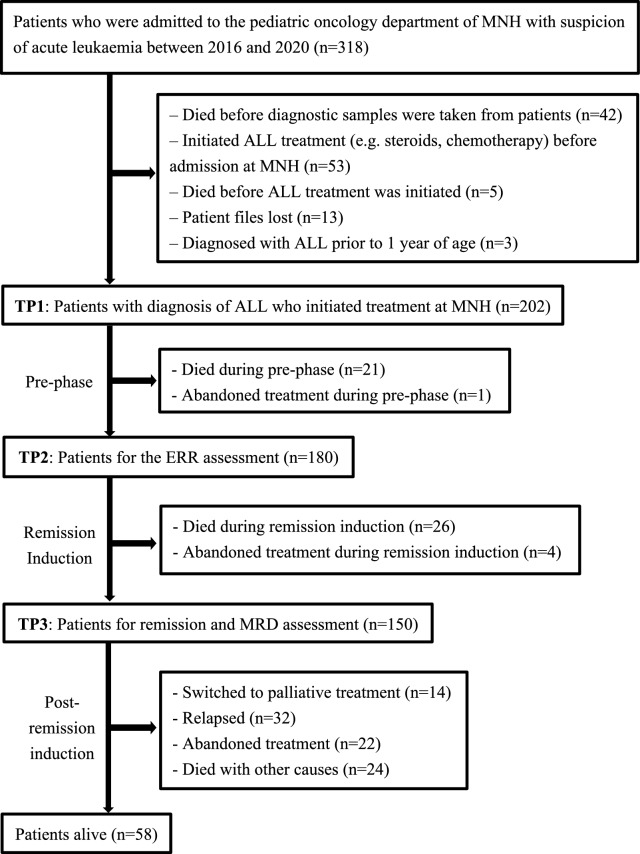
Flowchart describing the number of paediatric patients from MNH admission to post-induction treatment (MNH, Tanzania, 2016–2020). This flowchart describes the number of paediatric patients from MNH admission to post-remission induction treatment. Of 318 patients who were admitted to MNH, we enrolled 202 patients into this study. Of 202 patients, 180 patients received ERR assessment. After remission induction, 150 patients received remission and MRD assessment. Overall, 58 patients were event-free at the last date of follow-up. ALL, acute lymphoblastic leukaemia; ERR, early rapid responder; MNH, Muhimbili National Hospital; MRD, measurable residual disease

Descriptive statistics (percentages, medians) were used to describe patient characteristics, based on records with complete observations. EFS was estimated using the Kaplan–Meier method. Univariable and multivariable Cox proportional hazards models were used to estimate the effect of each prognostic factor on EFS. For these, we estimated the effects of prognostic factors from three different landmark times, each focused on patients alive at each timepoint: 1) timepoint (TP) 1: at treatment initiation, 2) TP2: at ERR assessment, and 3) TP3: at remission and MRD assessment (Fig. 2). The effect of ERR was analysed using TP2 and TP3; and the effect of remission was analysed using TP3, to avoid immortal-time bias. Analyses were performed on complete-case records and after multiple imputation of missing data (Appendix S1). To build multivariable Cox models, we selected prognostic factors based on previous literature and the results of univariable models run on complete-case records.

### Results

Between 2016 and 2020, 318 patients were admitted to the paediatric oncology ward of MNH with suspicion of acute leukaemia; and of these, 202 patients had confirmatory diagnosis of ALL, and initiated treatment at MNH (Fig. 2). We excluded 116 patients (116/318, 36%); including 42 who died before their diagnostic samples were taken, and 53 who initiated ALL treatment prior to MNH admission.

The median age at diagnosis was 6 years (range 1–19), and 42% were female (84/202) (Table 2). B- and T-lineages accounted for 70% (140/201) and 30% (61/201) of the leukaemias, respectively. Twenty-one percent of patients had central nervous system (CNS) involvement (30/140). Twelve percent of the male patients had testicular involvement (13/110). Sixty percent of the patients had positive hepatitis B surface antibody (HBsAb) (48/80). Patients were admitted at MNH after a median duration of 60 days since symptom onset (Supplementary Table S3, Supplementary Figure S1). Of 182 patients who were diagnosed at MNH, the median duration from admission to diagnosis was 7 days (range, 1–97) (Supplementary Table S3, Supplementary Figure S2). The median follow-up period was 41 months.

**Table 2:** Demographic and clinical characteristics of 202 pediatric patients with ALL (MNH, Tanzania, 2016 − 2020)

	*N*	(%)	Complete observations (*N*)†	Missing data (*N*)
Female	84	(41.6)	202	0
Age (years)			200	2
Median, range	6	(1,19)		
Patients 1–9 years	134	(67.0)		
Patients 10–19 years	66	(33.0)		
ALL lineage			201	1
B	140	(69.7)		
T	61	(30.4)		
Clinical presentation				
Splenomegaly	127	(65.1)	195	7
Hepatomegaly	109	(56.8)	192	10
Oedema	52	(26.1)	199	3
Comorbidities				
Sickle cell anaemia	15	(7.5)	201	1
HIV	1	(0.7)	148	54
Testicular involvement	13	(11.8)	110	8
CNS involvement	30	(21.4)	140	62
HBsAg serostatus positive	1	(0.7)	148	54
HBsAb serostatus positive	48	(60.0)	80	122
White blood cell ≥ 50 000/μL	52	(25.9)	201	1
NCI risk classification ‡			201	1
Standard	74	(36.8)		
High	127	(63.2)		
Initial regimen §			180	0
A	7	(3.9)		
B	156	(86.7)		
C	17	(9.4)		

^†^The proportions were calculated out of complete observations

^‡^Patients (age 1–9.99 years, initial white blood cell count < 50 000/μL) with B-ALL are considered standard risk. Patients (age ≥ 10 years, initial white blood cell count ≥ 50 000/μL) with B-ALL are considered high risk. All patients with T-ALL and those with CNS and testicular involvement are considered high risk

^§^This does not include 22 patients who died or abandoned treatment during pre-phase

Abbreviations: ALL, acute lymphoblastic leukaemia; CNS, central nervous system; HBsAb, hepatitis B surface antibody; HBsAg, hepatitis B surface antigen; HIV, human immunodeficiency virus; MNH, Muhimbili National Hospital; NCI, National Cancer Institute

**Fig. 3:**
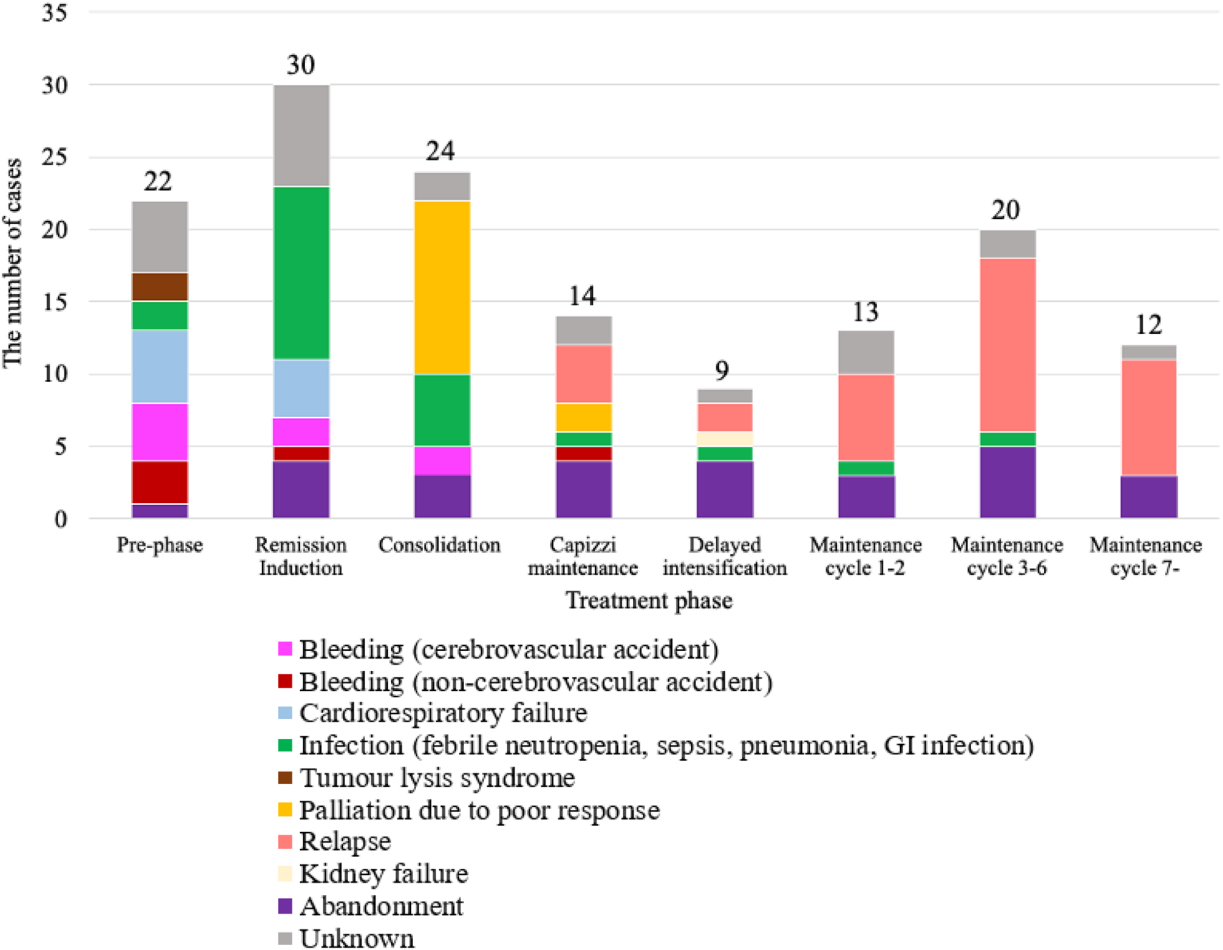
Clinical events of paediatric patients with ALL at different phases of chemotherapy (MNH, Tanzania, 2016–2020). The bar graph indicates the timing of clinical events among paediatric patients with ALL at MNH from 2016 to 2020. The major causes of death during pre-phase and remission induction were infections (*n* = 14), bleeding complications (*n* = 10), and cardiorespiratory failures (*n* = 9). Treatment abandonment was observed across the whole treatment. ALL, acute lymphoblastic leukaemia; GI, gastro-intestinal; MNH, Muhimbili National Hospital

Of the patients with available information, 41% of the patients achieved ERR after pre-phase (43/106) (Table 3). Of 142 patients for whom data could be confirmed at TP3, 126 (89%) achieved remission; of 126 patients who achieved remission, 76% of the patients achieved MRD negativity (96/126). Twenty-six percent of the patients (52/202) did not complete remission induction due to death (n = 47) and treatment abandonment (n = 5). The major causes of death during this period were infections [e.g., febrile neutropenia (FN)] (n = 14), bleeding complications (e.g., cerebral haemorrhage) (n = 10), and cardiorespiratory failures (n = 9), on top of 12 unknown causes of death (Fig. 3). During the entire treatment, 25% of the patients relapsed (32/126) and 13% of the patients abandoned treatment (27/202). Of 144 clinical events, 45 events occurred during the maintenance phase (31%, 45/144).

**Table 3:** Clinical outcomes of 202 paediatric patients with ALL (MNH, Tanzania, 2016–2020)

	*N*	(%)			Complete observations (*N*)†	Missing data (*N*)
Early rapid responder (ERR) ‡	43	(40.6)			106	74
Remission achieved after remission induction (RI) §	126	(88.7)			142	8
Remission achieved after RI (considering death or treatment abandonment before remission assessment as induction failures) ¶	126	(64.9)			194	8
Remission, MRD negative			96/126	(76.2)		
Remission, MRD positive			9/126	(7.1)		
Remission, MRD unknown			21/126	(16.7)		
Death or treatment abandonment before remission assessment	52	(25.7)			202	0
Treatment abandonment at any point of treatment	27	(13.4)			202	0
Relapse	32	(25.4)			126	0
Relapse involving CNS	14	(11.1)			126	0

ALL, acute lymphoblastic leukaemia; CNS, central nervous system; ERR, early rapid responder; MNH, Muhimbili National Hospital; MRD, measurable residual disease; RI, remission induction

^†^The proportions were calculated out of complete observations

^‡^This does not include 22 patients who died or abandoned treatment before ERR assessment

^§^This does not include 52 patients who died or abandoned treatment before remission and MRD assessment

^¶^This considers that patients who died or abandoned treatment before remission assessment did not achieve remission

The median EFS was 275 days (95% CI 179–469) and the 2-year EFS was 36% (95% CI 30–43) (Supplementary Figure S3). In multivariable Cox regression analysis performed on imputed data, patients with oedema had twice the hazard of clinical events compared to patients without oedema (Table 4). Non-ERRs had twice the hazard of clinical events compared to ERRs. Patients who did not achieve remission at the end of RI had five times the hazard of clinical events compared to those who achieved remission. At TP3, T-ALL had 1.8 times the hazard of clinical events compared to B-ALL, whereas at TP1 and TP2, CNS involvement had 1.9 times the hazard of clinical events compared to no CNS involvement. The univariable analysis on complete-case records showed similar results. (Supplementary Table S4).

**Table 4:** Multivariable Cox regression analysis on event-free survival of paediatric patients with ALL after multiple imputation (MNH, Tanzania, 2016–2020)

	At treatment initiation (*n* = 202)		After pre-phase (*n* = 180)		After remission induction (*n* = 150)	
	HR	95%CI	HR	95%CI	HR	95%CI
T-ALL vs. B-ALL (ref.)	1.40	0.95, 2.07	1.37	0.88, 2.12	1.77^*^	1.07, 2.93
Male vs. female (ref.)	0.84	0.59, 1.19	0.76	0.52, 1.13	0.76	0.48, 1.20
Age 10–19 years vs. 1–9 years (ref.)	0.91	0.62, 1.32	1.09	0.72, 1.64	1.03	0.64, 1.65
Oedema vs. no oedema (ref.)	2.11^***^	1.44, 3.12	2.07^**^	1.32, 3.22	2.62^***^	1.57, 4.39
WBC $\ge$ 50 000/$\mu$ L vs. WBC < 50 000/$\mu$ L (ref.)	1.27	0.86, 1.88	1.39	0.89, 2.17	1.24	0.73, 2.10
CNS involvement vs. No CNS involvement (ref.)	1.91^*^	1.13, 3.23	1.91^**^	1.18, 3.10	1.39	0.75, 2.57
Time from home to MNH > 2 h vs. $\le$ 2 h (ref.)	1.38	0.94, 2.03	1.23	0.80, 1.88	0.87	0.53, 1.42
Non-ERR vs. ERR (ref.)			2.11^*^	1.19, 3.72	2.04^*^	1.07, 3.89
Non-remission vs. remission (ref.)					5.74^***^	2.67, 12.35

ALL, acute lymphoblastic leukaemia; CI, confidence interval; CNS, central nervous system; ERR, early rapid responder; HR, hazard ratio; MNH, Muhimbili National Hospital; Non-ERR, non-early rapid responder; ref, reference; WBC, white blood cell

^*^p value $$\le$$ 0.05

^**^ *p* value $$\le$$ 0.01

^***^p value $$\le$$ 0.001

## Discussion

This study described the clinical characteristics and outcomes of paediatric ALL at a tertiary-level hospital in Tanzania. The proportions of T-lineage (30%), CNS involvement (21%) and testicular involvement (12%) were higher than those of HICs (T-lineage, 15%; CNS involvement 2–3%; testicular involvement 1–2%), which are risk factors for early mortality (Table 2) [10–12]. The development of a population-based leukaemia registry may provide further insights into the differences in clinical characteristics.

The seropositivity of HBsAb was 60% among those who were screened, despite a local report that the hepatitis B vaccine coverage is over 90% (Table 2) [13]. The hepatitis B vaccine was included in the routine immunization program for infants in 2002, and since 2009 has been given as a pentavalent vaccine together with diphtheria, pertussis, tetanus, and *Haemophilus influenza* type B [14]. Forty percent of children had inadequate immunity against hepatitis B, possibly due to their birth before the introduction of the national immunization program, incompletion of three doses of the vaccine, or lack or loss of an immune response to the vaccine [15]. Our results underline the importance of screening patients for hepatitis B serostatus upon hospital admission; specifically, because patients with leukaemia often require blood transfusion, which exposes them to a risk of hepatitis B transmission (hepatitis B prevalence estimated to be 7% in Tanzania), and those with hepatitis B infection should be monitored for virus reactivation during treatment [16]. On top of infant vaccination, WHO recommends routine hepatitis B screening with subsequent treatment for pregnant women and administration of hepatitis B vaccine within 24 h of birth to prevent mother-to-child transmission [17]. These strategies have not been fully implemented in Tanzania. [18]

We observed a delay in patient presentation and leukaemia diagnosis (Supplementary Table S3, Supplementary Figures S1, S2). Forty-two patients who were initially suspected of leukaemia died before their diagnostic samples were taken (Fig. 2). In resource-limited settings, pre-diagnostic deaths can be the result of delayed presentation; and accompanied by malnutrition, infection, and other comorbidities. A study from a tertiary-level hospital in western Kenya reports that lack of education of primary care physicians in peripheral centres may contribute to low referral rate and missed diagnoses of childhood leukaemia [19]. A qualitative study with interviews of patients, medical staff, and policymakers is necessary to map patient journeys, identify barriers to timely diagnosis, and to develop effective interventions based on the local context.

A quarter of patients died or abandoned treatment before remission assessment (26%, 52/202) (Table 3). The two major causes of deaths during this period were infections and bleeding complications. They are common causes of early deaths in both HICs and LMICs, although the proportions of induction deaths may vary significantly between 1 and 24%. [20–22] Blood cultures were not routinely taken at the ward, and we had incomplete information on the species and the antibiotic sensitivity of the bacteria. We often experienced delays in giving broad-spectrum antibiotics to patients with FN due to miscommunication among medical staff and insufficient adherence to FN escalation protocol. The substandard quality of generic antibiotics could also be of concern. Infection deaths could be prevented by adopting a pediatric early warning score at the ward, re-evaluating the safe and accurate pathways from a doctor’s prescription to drug administration, the immediate administration of anti-pseudomonal antibiotics, and practicing infection control measures and antimicrobial stewardship. A removal of anthracycline from induction treatment could be considered for National Cancer Institute (NCI) standard-risk patients to reduce myelosuppression, although the information on genetic mutations was not available [23, 24]. Some bleeding complications may be avoidable through monitoring platelet counts and coagulation markers, as well as by providing platelet transfusions. However, the blood products, including whole blood transfusion and platelet concentrates are not always readily available in Tanzania; and factors beyond platelet counts are known to affect bleeding in the paediatric population. [25]

We observed a high number of relapse cases (25%, 32/126), especially during the maintenance phase; in part because of frequent interruptions and discontinuation of the treatment, including due to severe infections such as FN. Adjustment of treatment intensity may shorten the period of interruptions. Other potential reasons for relapses include: 1) the rare suspension of L-asparaginase for those with severe allergic reactions, 2) substandard quality of L-asparaginase, and 3) missed oral medications such as dexamethasone or 6-mercaptopurine in the outpatient setting [26, 27]. Inadequate dose of asparaginase is associated with inferior disease-free survival in NCI high-risk patients with B-ALL. Patients missed outpatient appointments due to financial or physical barriers, or because of poor communication between healthcare professionals and family members. A study from Malawi suggested that a comprehensive package of free services including transportation, accommodation and treatment, and active follow-up of outpatients may decrease treatment abandonment [28]. Cytogenetic and molecular information may shed light on the identification of high-risk patients.

The multivariable analysis identified ERR, remission, and oedema as significant prognostic factors for event-free survival (Table 4). Good response to corticosteroids and remission at the end of RI are known prognostic factors in paediatric ALL [29, 30]. Assessment of the treatment response was also useful in risk stratifying the paediatric ALL population; thus allowing clinicians to reduce the treatment intensity among the low-risk population [31]. We were not able to ascertain the anatomical location and the mechanism of oedema from patient records; oedema may have reflected comorbidities, such as multi-organ failure or malnutrition.

In this cohort, the prognosis for female patients is equivalent to that for male patients, or even seems to be poorer (Table 4, Supplementary Figure S4). This result contradicts the previous literature indicating that male patients have higher risks of leukaemia death than female patients [32]. We observed higher proportions of T-lineage (male 36% vs. female 22%) and NCI high-risk (male 68% vs. female 57%) among male patients, but these risks were not reflected in the EFS. Female patients had higher proportions of palliation (female 11% vs. male 4%) and treatment abandonment (female 15% vs. male 12%) than male patients, which may have contributed to their inferior survival. We need to further evaluate this effect after increasing the sample size and calculating their durations of treatment discontinuation and interruptions.

The comparison between the current and previous cohorts of patients showed that more patients currently received L-asparaginase (24 in 2008 to 2010, 63 in 2011 to 2013, 180 in 2016 to 2020) and had documentation of the cerebrospinal fluid (CSF) analysis (20% in 2011 to 2013, 69% in 2016 to 2020) (Supplementary Table S5). [8, 33] These are partial indicators of the improved medical infrastructure. There was no difference in 2-year EFS (33% in 2008 to 2010, 31% in 2011 to 2013 and 36% in 2016 to 2020). In the past, patients who reached the hospital and received treatment were highly selected, whereas the number of patients seen at the ward doubled since 2010 (27/year to 55/year in 2020). A modest improvement in 2-year EFS may not accurately reflect the improved performance at the paediatric oncology ward.

This study had several limitations. We excluded 53 patients who had confirmed ALL diagnosis, but received treatment before MNH admission. Tanzania is a large country, and it may take more than a day for patients to travel from their homes to MNH in Dar es Salaam. If patients had suspicious or confirmed diagnoses of ALL, steroids were initiated at peripheral hospitals to gain time to transfer them to MNH. Such patients were excluded from the survival analysis due to insufficient information on the duration and the content of their treatment before their arrival at MNH. However, a comparison of survival curves—prone-to-immortal-time bias due to the non-alignment of start of follow-up between patients—shows that their pattern of survival matches that of patients who started treatment at MNH (Supplementary Figure S5). It is important to ensure that patients at peripheral hospitals are swiftly and safely transferred to core facilities that can provide induction treatment. Due to the retrospective design of the study, a large fraction of data was lacking for variables, such as CSF analysis, ERR assessment, and HBsAb serostatus. We performed both complete-case analysis and multiple imputation to account for the missing data. Despite some suggestions about the non-proportionality of hazards for age and sex, this was not confirmed by Schoenfeld residuals and the inclusion of an interaction term with time in the Cox models.

## Conclusion

The number of new ALL admissions at MNH has doubled in the past decade. The prevention of early deaths and the proactive follow-up of patients throughout treatment are critical for improving the long-term survival of ALL in Tanzania.

## Supplementary Information

Supplementary material 1

## Data Availability

The data that supports the findings of this study are available from the corresponding author upon reasonable request.

## Abbreviations

ALL: Acute lymphoblastic leukaemia
BM: Bone marrow
CNS: Central nervous system
CSF: Cerebrospinal fluid
EFS: Event-free survival
ERR: Early rapid responder
FN: Febrile neutropenia
GI: Gastro-intestinal
HBsAb: Hepatitis B surface antibody
HIC: High-income country
HIV: Human immunodeficiency virus
HR: Hazard ratio
LMIC: Low- and middle-income country
MNH: Muhimbili National Hospital
MRD: Measurable residual disease
NCI: National Cancer Institute
RI: Remission induction
TP: Time point
WBC: White blood cell
WHO: World Health Organization

## References

[1] Steliarova-Foucher E, Colombet M, Ries LAG, et al. International incidence of childhood cancer, 2001–10: a population-based registry study. Lancet Oncol. 2017;18(6):719–31. 10.1016/S1470-2045(17)30186-9. 28410997 10.1016/S1470-2045(17)30186-9 PMC5461370

[2] Ward E, DeSantis C, Robbins A, Kohler B, Jemal A. Childhood and adolescent cancer statistics, 2014. CA Cancer J Clin. 2014;64(2):83–103. 10.3322/caac.21219. 24488779 10.3322/caac.21219

[3] Hunger SP, Lu X, Devidas M, et al. Improved survival for children and adolescents with acute lymphoblastic leukemia between 1990 and 2005: a report from the children’s oncology group. J Clin Oncol. 2012;30(14):1663–9. 10.1200/JCO.2011.37.8018. 22412151 10.1200/JCO.2011.37.8018 PMC3383113

[4] Allemani C, Matsuda T, Carlo VD, et al. Global surveillance of trends in cancer survival 2000–14 (CONCORD-3): analysis of individual records for 37513025 patients diagnosed with one of 18 cancers from 322 population-based registries in 71 countries. Lancet. 2018;391(10125):1023–75. 10.1016/S0140-6736(17)33326-3. 29395269 10.1016/S0140-6736(17)33326-3 PMC5879496

[5] World Health Organization. WHO global initiative for childhood cancer: an overview. https://www.who.int/docs/default-source/documents/health-topics/cancer/who-childhood-cancer-overview-booklet.pdf. Accessed August 28, 2024.

[6] Howard SC, Davidson A, Luna-Fineman S, et al. A framework to develop adapted treatment regimens to manage pediatric cancer in low- and middle-income countries: the pediatric oncology in developing countries (PODC) committee of the international pediatric oncology society (SIOP). Pediatr Blood Cancer. 2017;64(Suppl):5. 10.1002/pbc.26879. 10.1002/pbc.26879 29297619

[7] Howard SC, Pedrosa M, Lins M, et al. Establishment of a pediatric oncology program and outcomes of childhood acute lymphoblastic leukemia in a resource-poor area. JAMA. 2004;291(20):2471–5. 10.1001/jama.291.20.2471. 15161898 10.1001/jama.291.20.2471

[8] Kersten E, Scanlan P, Dubois SG, Matthay KK. Current treatment and outcome for childhood acute leukemia in Tanzania. Pediatr Blood Cancer. 2013;60(12):2047–53. 10.1002/pbc.24576. 24039163 10.1002/pbc.24576

[9] Vora A, Goulden N, Wade R, et al. Treatment reduction for children and young adults with low-risk acute lymphoblastic leukaemia defined by minimal residual disease (UKALL 2003): a randomized controlled trial. Lancet Oncol. 2013;14(3):199–209. 10.1016/S1470-2045(12)70600-9. 23395119 10.1016/S1470-2045(12)70600-9

[10] Hunger SP, Mullighan CG. Acute lymphoblastic leukemia in children. N Engl J Med. 2015;373(16):1541–52. 10.1056/NEJMra1400972. 26465987 10.1056/NEJMra1400972

[11] Thastrup M, Duguid A, Mirian C, Schmiegelow K, Halsey C. Central nervous system involvement in childhood acute lymphoblastic leukemia: challenges and solutions. Leukemia. 2022;36(12):2751–68. 10.1038/s41375-022-01714-x. 36266325 10.1038/s41375-022-01714-x PMC9712093

[12] Nguyen HTK, Terao MA, Green DM, Pui CH, Inaba H. Testicular involvement of acute lymphoblastic leukemia in children and adolescents: diagnosis, biology, and management. Cancer. 2021;127(17):3067–81. 10.1002/cncr.33609. 34031876 10.1002/cncr.33609 PMC9677247

[13] World Health Organization. Immunization United Republic of Tanzania 2023 country profile. https://cdn.who.int/media/docs/default-source/country-profiles/immunization/2023-country-profiles/immunization_tza_2023.pdf?sfvrsn=9fbc3b6d_3&download=true. Accessed November 20, 2023.

[14] The United Republic of Tanzania, Ministry of Health and Social Welfare—Tanzania Mainland. Expanded programme on immunization 2010–2015 comprehensive multi year plan. https://bidinitiative.org/wp-content/uploads/1405554135TanzaniaComprehensivemultiyearplanfor20102015Year2011.pdf. Accessed November 20, 2023.

[15] Metodi J, Aboud S, Mpembeni R, Munubhi E. Immunity to hepatitis B vaccine in Tanzanian under-5 children. Ann Trop Paediatr. 2010;30(2):129–36. 10.1179/146532810X12703902516167. 20522299 10.1179/146532810X12703902516167

[16] Schweitzer A, Horn J, Mikolajczyk RT, Krause G, Ott JJ. Estimations of worldwide prevalence of chronic hepatitis B virus infection: a systematic review of data published between 1965 and 2013. Lancet. 2015;386(10003):1546–55. 10.1016/S0140-6736(15)61412-X. 26231459 10.1016/S0140-6736(15)61412-X

[17] World Health Organization. Global health sector strategy on viral hepatitis 2016–2021. Towards ending viral hepatitis. https://www.who.int/publications/i/item/WHO-HIV-2016.06. Accessed April 17, 2025.

[18] Kilonzo SB, Gunda DW, Mpondo BCT, Bakshi FA, Jaka H. Hepatitis B virus infection in Tanzania: current status and challenges. J Trop Med. 2018. 10.1155/2018/4239646. 29666656 10.1155/2018/4239646 PMC5831599

[19] Severance TS, Njuguna F, Olbara G, et al. An evaluation of the disparities affecting the underdiagnosis of pediatric cancer in Western Kenya. Pediatr Blood Cancer. 2022;69(10): e29768. 10.1002/pbc.29768. 35593641 10.1002/pbc.29768

[20] Ehrlich BS, McNeil MJ, Pham LTD, et al. Treatment-related mortality in children with cancer in low-income and middle-income countries: a systematic review and meta-analysis. Lancet Oncol. 2023;24(9):967–77. 10.1016/S1470-2045(23)00318-2. 37517410 10.1016/S1470-2045(23)00318-2 PMC10812862

[21] Seif AE, Fisher BT, Li Y, et al. Patient and hospital factors associated with induction mortality in acute lymphoblastic leukemia. Pediatr Blood Cancer. 2014;61(5):846–52. 10.1002/pbc.24855. 24249480 10.1002/pbc.24855 PMC3951664

[22] Olbara G, van der Wijk T, Njuguna F, et al. Childhood acute lymphoblastic leukemia treatment in an academic hospital in Kenya: treatment outcomes and health-care providers’ perspectives. Pediatr Blood Cancer. 2021;68(12): e29366. 10.1002/pbc.29366. 34569156 10.1002/pbc.29366

[23] Smith M, Arthur D, Camitta B, et al. Uniform approach to risk classification and treatment assignment for children with acute lymphoblastic leukemia. J Clin Oncol. 1996;14(1):18–24. 10.1200/JCO.1996.14.1.18. 8558195 10.1200/JCO.1996.14.1.18

[24] Rujkijyanont P, Inaba H. Diagnostic and treatment strategies for pediatric acute lymphoblastic leukemia in low- and middle-income countries. Leukemia. 2024;38(8):1649–62. 10.1038/s41375-024-02277-9. 38762553 10.1038/s41375-024-02277-9

[25] Josephson CD, Granger S, Assmann SF, et al. Bleeding risks are higher in children versus adults given prophylactic platelet transfusions for treatment-induced hypoproliferative thrombocytopenia. Blood. 2012;120(4):748–60. 10.1182/blood-2011-11-389569. 22538854 10.1182/blood-2011-11-389569 PMC3462047

[26] Gupta S, Wang C, Raetz EA, et al. Impact of asparaginase discontinuation on outcome in childhood acute lymphoblastic leukemia: a report from the children’s oncology group. J Clin Oncol. 2020;38(17):1897–905. 10.1200/JCO.19.03024. 32275469 10.1200/JCO.19.03024 PMC7280050

[27] Bhatia S, Landier W, Shangguan M, et al. Nonadherence to oral mercaptopurine and risk of relapse in Hispanic and non-Hispanic white children with acute lymphoblastic leukemia: a report from the children’s oncology group. J Clin Oncol. 2012;30(17):2094–101. 10.1200/JCO.2011.38.9924. 22564992 10.1200/JCO.2011.38.9924 PMC3601449

[28] Chakumatha E, Khofi H, Landman L, et al. Towards zero percent treatment abandonment of patients with common and curable childhood cancer types in Blantyre, Malawi. Pediatr Blood Cancer. 2022;69(12): e29899. 10.1002/pbc.29899. 35869892 10.1002/pbc.29899

[29] Dördelmann M, Reiter A, Borkhardt A, et al. Prednisone response is the strongest predictor of treatment outcome in infant acute lymphoblastic leukemia. Blood. 1999;94(4):1209–17. 10438708

[30] Silverman LB, Gelber RD, Young ML, Dalton VK, Barr RD, Sallan SE. Induction failure in acute lymphoblastic leukemia of childhood. Cancer. 1999;85(6):1395–404. 10.1002/(sici)1097-0142(19990315)85:6%3c1395::aid-cncr25%3e3.0.co;2-2. 10189148 10.1002/(sici)1097-0142(19990315)85:6<1395::aid-cncr25>3.0.co;2-2

[31] Pedrosa F, Coustan-Smith E, Zhou Y, et al. Reduced-dose intensity therapy for pediatric lymphoblastic leukemia: long-term results of the Recife RELLA05 pilot study. Blood. 2020;135(17):1458–66. 10.1182/blood.2019004215. 32027741 10.1182/blood.2019004215 PMC7180080

[32] Pui CH, Boyett JM, Relling MV, et al. Sex differences in prognosis for children with acute lymphoblastic leukemia. J Clin Oncol. 1999;17(3):818–24. 10.1200/JCO.1999.17.3.818. 10071272 10.1200/JCO.1999.17.3.818

[33] Cohler C, Jumanne S, Kaijage J, DuBois SG, Scanlan P, Matthay KK. Evaluation and outcome of central nervous system involvement in pediatric acute lymphoblastic leukemia in Dar es Salaam. Tanzania Pediatr Blood Cancer. 2016;63(3):458–64. 10.1002/pbc.25829. 26529141 10.1002/pbc.25829

