# Supplementary material for: Clinical characteristics and outcomes of paediatric acute lymphoblastic leukaemia in a tertiary hospital in Tanzania: a single-centre observational study
Source: Trop Med Health. 2025 May 27;53:76. doi: 10.1186/s41182-025-00760-2 (PMC12107958; doi:10.1186/s41182-025-00760-2)
Supplement: Supplementary file 1 — Supplementary material 1 [file 41182_2025_760_MOESM1_ESM.docx]

**Supplementary information**

Appendix S1 Methods of multiple imputation.

We applied multiple imputation by chained equations to estimate the likely values of all variables with missing information, therefore using all 202, 180 and 150 patients who were alive at each landmark time.^1^ We constructed 30 imputed datasets and combined the results using Rubin’s rules.

Reference

1. StataCorp. Stata 18 multiple-imputation reference manual. College Station, TX: Stata Press; 2023.

Supplementary Table S1 Facilities and available resources at the paediatric oncology ward of MNH, Tanzania.

| Resource | Availability |
| --- | --- |
| Diagnostic |  |
| X-ray | Consistently available at MNH |
| CT, MRI | Consistently available at MNH |
| Complete blood count | Consistently available at MNH, daily |
| Chemistry panel | Consistently available at MNH, daily |
| Coagulation studies | Consistently available at MNH, daily |
| Peripheral blood morphology | Consistently available at MNH, with 1-3 weeks delay |
| Bone marrow morphology | Consistently available at MNH, with 1-3 weeks delay |
| Immunohistochemistry | Consistently available at MNH with limited access |
| Flow cytometry | Intermittently available at MNH or samples airfreight to Dublin |
| Cytogenetics | Not available |
| CSF cytology | Consistently available, with 1-3 weeks delay |
| Therapeutic, supportive |  |
| Whole blood transfusions | Consistently available at MNH, with delay of several hours to few days |
| Platelet transfusions | Intermittently available at MNH, with delay of several hours to several days |
| Fresh frozen plasma | Intermittently available at MNH |
| Meropenem | Consistently available at MNH, free of charge through charitable donation |
| Piperacillin/tazobactam | Consistently available at MNH, free of charge through charitable donation |
| Vancomycin | Consistently available at MNH, free of charge through charitable donation |
| Gentamicin | Consistently available at MNH, free of charge through charitable donation |
| Ciprofloxacin | Consistently available at MNH, free of charge through charitable donation |
| Itraconazole | Consistently available at MNH, free of charge through charitable donation |
| Fluconazole | Consistently available at MNH, free of charge through charitable donation |
| Amphotericin B | Consistently available at MNH, free of charge through charitable donation |
| Trimethoprim/sulfamethoxazole | Consistently available at MNH, free of charge through charitable donation |
| G-CSF | Consistently available at MNH, free of charge through charitable donation |
| Therapeutic, cancer care |  |
| Bone marrow transplant | Not available |
| Radiation therapy | Consistently available at nearby facility – the Ocean Road Cancer Institute, free of charge |
| Staffing |  |
| Nursing: patient ratio | 1:8 during daytime, 1:11 at night |
| Paediatric oncologist | Three available for entire paediatric oncology ward at MNH |
| General paediatrician | Three available for entire paediatric oncology ward at MNH |
| General practitioner | Five available for entire paediatric oncology ward at MNH |
| Paediatric oncology fellow | Six available for entire paediatric oncology ward at MNH |
| Social worker | Four available for entire children’s ward at MNH |
| Nutritionist | One available for entire children’s ward at MNH |
| Facilities |  |
| Paediatric ICU | Available at MNH |
| Housing for caregiver/family | Available at MNH |
| Chemotherapy |  |
| All products for Regimen A, B and C | Consistently available at MNH, free of charge through charitable donation |

Abbreviations: CSF, cerebrospinal fluid; CT, computed tomography; G-CSF, granulocyte-colony stimulating factor; ICU, intensive care unit; MNH, Muhimbili National Hospital; MRI, magnetic resonance imaging.

Supplementary Table S2 Chemotherapy protocol of paediatric acute lymphoblastic leukaemia at MNH, Tanzania.

| Pre-phase | Remission induction | Consolidation | Capizzi maintenance | Delayed intensification | Maintenance |
| --- | --- | --- | --- | --- | --- |
| PSL 40 mg/m^2^ on day 1–7 accompanied by TLS prophylaxis  PSL 40 mg/m^2^ on day 1–7 accompanied by TLS prophylaxis | **Remission Induction A**  **DEXA 6 mg/m^2^/day PO in 2 divided doses for 21 days and tapered over the next 7 days**  VCR 1.5 mg/m^2^ IV on days 2,9,16,23,30  **L-asp 20 000 units/m^2^ IM on days 1,3,5,7,10,12,22,24,26,29,31,33 (12 doses)** †  **6-MP 75 mg/m^2^ PO starting on day 29 (week 6) and continue for 4 weeks (week 9)**  Triple IT 3 times by the end of this phase ‡ | **Consolidation A**  **6-MP 75 mg/m^2^ PO until end of week 9**  Triple IT (3 times) ‡ | VCR 1.5 mg/m^2^ IV on days 2,12,22,32,42  MTX 100 mg/m^2^ IV on day 2. Escalate subsequent doses by 50 mg/m^2^ to the maximum of 300 mg/m^2^ if toxicity allows, on days 12,22,32,42.  L-asp 20 000 units/m^2^ IM on days 3,5,7,9,11,13,25,27,29,31,33,35 (12 doses) †  Triple IT (2 times) ‡ | DEXA 10 mg/m^2^/day PO on days 2–8,16–22  VCR 1.5 mg/m^2^ IV on days 2,9,16  DOX 25 mg/m^2^ IV on days 2,9,16.  L-asp 20 000 units/m^2^ IM on days 4,6,8,10,12,14 (6 doses) †  Cy 1 g/m^2^ IV on day 29  6-MP 60 mg/m^2^ PO from days 29-42  Ara-C 75 mg/m^2^/day IV on days 30–33,37–40 (8 doses)  Triple IT (3 times) ‡ | 12 weeks = 1 cycle  DEXA 6 mg/m^2^/day PO on days 1–5, 29–33, 57–61  VCR 1.5 mg/m^2^ IV on days 1,29,57  6-MP 75 mg/m^2^/day PO throughout maintenance  MTX 20 mg/m^2^ PO on days 1,8,22,29,36,43,50,57,64,71,78  Triple IT on day 15 ‡ |
|  | Weeks 2–6 | Weeks 7–9 | Weeks 10–17 | Weeks 18–24 | Weeks 25–120 (girls)  Weeks 25–168 (boys) |
|  | **Remission Induction B**  **DEXA 6 mg/m^2^/day PO in 2 divided doses for 21 days and tapered over the next 7 days**  VCR 1.5 mg/m**^2^** IV on days 2,9,16,23,30  **DNR 25 mg/m^2^ IV on days 2,9,16,23**  **L-asp 20 000 units/m^2^ IM on days 1,3,5,7,10,12,22,24,26,29,31,33 (12 doses)** †  **6-MP 60 mg/m^2^ PO starting on day 29 (week 6) and continue for 5 weeks (week 10)**  Triple IT 3 times by the end of this phase ‡ | **Consolidation B**  **6-MP 60 mg/m**^2^ **PO until end of week 10**  **Cy 1g /m^2^ IV on days 1,15**  **Ara-C 75 mg/m^2^/day IV in days 2–5,9–12,16–19,23–26 (16 doses)**  Triple IT (3 times) ‡ | Same as above. | Same as above. | Same as above. |
|  | Weeks 2–6 | Weeks 7–11 | Weeks 12–19 | Weeks 20–26 | Weeks 27–122 (girls)  Weeks 27–170 (boys) |
|  | **Remission Induction C**  **DEXA 6 mg/m**^2^**/day in 2 divided doses for 28 days and tapered over the next 7 days**  VCR 1.5 mg/m^2^ IV on day 2,9,16,23,30  **DNR 25 mg/m^2^ IV on days 2,9 and 45mg/m^2^ IV on days 16,23**  **L-asp 20 000 units/m^2^ IM on days 1,3,5,7,10,12,22,24,26,29,31,33 (12 doses)** †  **6-MP 60 mg/m^2^ PO starting on day 29 (week 6) and continue for 3 weeks (week 8)**  Triple IT 3 times by the end of this phase ‡ | **Consolidation C**  **6-MP 60 mg/m^2^ PO until end of week 8**  **6-MP 60 mg/m^2^ from week 11 until end of week 12**  **Cy 1 g/m^2^ IV on days 1,29**  **Ara-C 75 mg/m**^2^**/day IV in days 2–5,9–12,30–33,37–40 (16 doses)**  **VCR 1.5 mg/m^2^ IV on days 16,23, 44,51**  **L-asp 20 000 units/m^2^ IM on days 16,18,20,22,24,26,44,46,48,50,52,54 (12 doses)** †  Triple IT (3 times) ‡ | Same as above. | Same as above. | Same as above. |
|  | Weeks 2­–6 | Weeks 7–15 | Weeks 16–23 | Weeks 24–30 | Weeks 31–126 (girls)  Weeks 31–174 (boys) |

Bold fonts indicate treatment that differs by regimen.

† The dose of L-asparaginase was modified to 10 000 units/m^2^ in February 2020.

‡ Triple IT: < ages 2 years MTX 8mg, Ara-C 24mg, Hydrocortisone 16mg. ages 2-3 years MTX 10mg, Ara-C 30mg, Hydrocortisone 20mg. > ages 3 years MTX 12mg, Ara-C 36mg, Hydrocortisone 24mg.

Abbreviations: Ara-C, cytarabine; Cy, cyclophosphamide; DEXA, dexamethasone; DNR, daunorubicin; DOX, doxorubicin; IM, intramuscular; IT, intrathecal; IV, intravenous; L-asp, L-asparaginase; MNH, Muhimbili National Hospital; 6-MP, 6-Mercaptopurine; MTX, methotrexate; PO, per os; PSL, prednisolone; TLS, tumour lysis syndrome; VCR, vincristine.

Supplementary Table S3 Factors related to pre-hospital and post-hospital delays among paediatric patients with ALL (MNH, Tanzania, 2016–2020).

|  | N (%) | Complete observations (N) † | Missing data (N) |
| --- | --- | --- | --- |
| *Pre-hospital factors* |  |  |  |
| Treatment given at previous hospitals before MNH ‡ | 144 (83.2) | 173 | 29 |
| Blood transfusion | 99 (57.2) |  |  |
| Antibiotics | 78 (45.1) |  |  |
| Anti-tuberculosis medications | 18 (10.4) |  |  |
| Anti-malaria | 6 (3.5) |  |  |
| Days from symptom onset to the first hospital (days) – median, IQR § | 30 (21, 90) | 134 | 68 |
| Days from symptom onset to MNH (days) – median, IQR § | 60 (30, 93) | 196 | 6 |
| Patients living within 2 hours from home to MNH | 62 (31.3) | 198 | 4 |
| *Post-hospital factors* ¶ |  |  |  |
| Days from admission to diagnosis – median, range | 7 (1, 97) | 182 | 0 |
| Diagnostic delay (>7 days after hospital admission) ‡ | 84 (46.2) | 182 | 0 |
| Delay due to initial admission to another ward | 37/84 (44.1) |  |  |
| Awaiting flow cytometry result | 33/84 (39.3) |  |  |
| Awaiting histopathology result | 10/84 (11.9) |  |  |
| Peripheral blood revealed no abnormality | 10/84 (11.9) |  |  |
| Wrong initial diagnosis | 9/84 (10.7) |  |  |
| Days from diagnosis to treatment – median, range | 0 ($-$28, 20) | 182 | 0 |
| Days from admission to treatment – median, range | 6 (0, 98) | 182 | 0 |
| Treatment initiated before confirmed diagnosis | 53 (29.1) | 182 | 0 |

† The proportions were calculated out of complete observations.

‡ Treatments at previous hospitals and reasons for diagnostic delay could be more than one.

§ The symptom onset was defined as the date when a parent first noticed the child’s sick status irrespective of the child’s symptom. The symptom of the child may or may not have been truly related to leukaemia.

¶ The denominator (n=182) included those who were diagnosed at MNH.

Abbreviations: ALL, acute lymphoblastic leukaemia; IQR, interquartile range; MNH, Muhimbili National Hospital.

Supplementary Table S4 Univariable Cox regression analysis on event-free survival of paediatric patients with ALL by complete-case analysis (MNH, Tanzania, 2016–2020).

|  | At treatment initiation (n=131) | | After prephase  (n=84) | | After remission induction (n=72) | |
| --- | --- | --- | --- | --- | --- | --- |
|  | HR | 95%CI | HR | 95%CI | HR | 95%CI |
| T-ALL vs. B-ALL (ref.) | 1.43 | 0.90, 2.26 | 1.37 | 0.75, 2.50 | 1.08 | 0.54, 2.15 |
| Male vs. Female (ref.) | 0.75 | 0.48, 1.16 | 0.67 | 0.39, 1.17 | 0.71 | 0.39, 1.29 |
| Age 10 to 19 years vs. 1 to 9 years (ref.) | 1.42 | 0.91, 2.21 | 1.06 | 0.60, 1.87 | 1.16 | 0.63, 2.15 |
| Oedema vs. No oedema (ref.) | 1.84^*^ | 1.13, 3.02 | 2.49^**^ | 1.41, 4.38 | 2.04^*^ | 1.09, 3.84 |
| WBC$\geq$50 000/$\mu$L vs. WBC<50 000/$\mu$L (ref.) | 1.49 | 0.93, 2.37 | 1.04 | 0.56, 1.96 | 1.12 | 0.57, 2.23 |
| CNS involvement vs. No CNS involvement (ref.) | 1.53 | 0.92, 2.53 | 0.78 | 0.37, 1.67 | 0.84 | 0.37, 1.90 |
| Time from home to MNH>2 hours vs. $\leq$2 hours (ref.) | 1.35 | 0.85, 2.15 | 1.35 | 0.73, 2.50 | 1.49 | 0.76, 2.91 |
| Non-ERR vs. ERR (ref.) |  |  | 2.51^**^ | 1.34, 4.72 | 2.46^**^ | 1.24, 4.90 |
| Non-remission vs. Remission (ref.) |  |  |  |  | 3.78^**^ | 1.66, 8.58 |

*p-value$\leq$0.05

** p-value$\leq$0.01

Abbreviations: ALL, acute lymphoblastic leukaemia; CI, confidence interval; CNS, central nervous system; ERR, early rapid responder; HR, hazard ratio; MNH, Muhimbili National Hospital; Non-ERR, non-early rapid responder; WBC, white blood cell.

Supplementary Table S5 Comparison of clinical characteristics and outcomes between paediatric patients with ALL who were admitted at MNH from 2008 to 2010, 2011 to 2013 and 2016 to 2020.

|  | N † | 2008–2010 ‡ | N † | 2011–2013 ‡ | N † | 2016–2020 |
| --- | --- | --- | --- | --- | --- | --- |
| ALL lineage – no. (%) | 81 |  | 156 |  | 202 |  |
| Unknown |  | 81 (100) |  | 114 (73) |  | 1 (1) |
| B |  |  |  | 23 (15) |  | 140 (69) |
| T |  |  |  | 19 (12) |  | 61 (30) |
| NCI risk classification – no. (%) |  |  | 156 |  | 201 |  |
| Standard |  |  |  | 64 (41) |  | 74 (37) |
| High |  |  |  | 92 (59) |  | 127 (63) |
| Received L-asparaginase – no. |  | 24 |  | 63 |  | 180 |
| Treatment abandonment – no. (%) | 81 | 7 (9) | 156 | 15 (10) | 202 | 27 (13) |
| 2-year EFS – % (95%CI) § |  | 33  (21–47) |  | 31  (23–39) |  | 36  (30–43) |
| Documentation of CSF analysis – no. (%) |  |  | 128 | 25 (20) | 202 | 140 (69) |

† The denominator represents number with complete observations.

‡ Data derived from two publications, Kersten E, et al. Pediatr Blood Cancer. 2013;60(12):2047-53, and Cohler C, et al. Pediatr Blood Cancer. 2016;63(3):458-64.

§ Careful consideration is required for the comparison because the baseline characteristics, inclusion/exclusion criteria, definition of events, date of data entry, and the follow-up periods for the three cohorts are not entirely equivalent.

Abbreviations: ALL, acute lymphoblastic leukaemia; CI, confidence interval; CSF, cerebrospinal fluid; EFS, event-free survival; MNH, Muhimbili National Hospital; NCI, National Cancer Institute.

Supplementary Figure S1 Event-free survival of paediatric patients with ALL grouped by the duration from symptom onset to MNH admission, n=196 (MNH, Tanzania, 2016–2020).


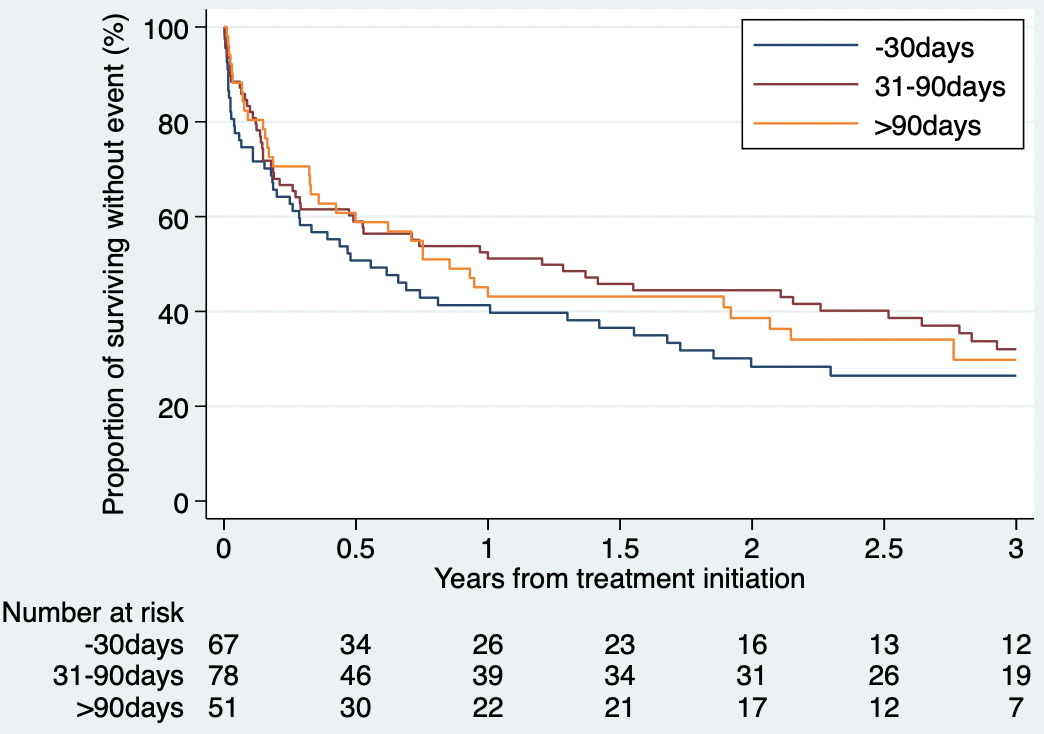


Log-rank test, p=0.42

The Kaplan-Meier curve describes the EFS of paediatric patients who initiated treatment for ALL at MNH from January 1, 2016 to December 31, 2020, grouped by duration from symptom onset to MNH admission. The information was available for 196 patients.

The proportions of clinical events before the end of remission induction (RI) were 28% (19/67) for those admitted within 30 days, 23% (18/78) for those admitted between 31 to 90 days, and 25% (13/51) for those admitted after 91 days. There was no evidence of an association between the duration from symptom onset to MNH admission and clinical events before the end of RI (p-value=0.80, Fisher’s exact test). The difference in the EFS was not statistically significant (Log-rank test, p=0.42).

Abbreviations: ALL, acute lymphoblastic leukaemia; EFS, event-free survival; MNH, Muhimbili National Hospital; RI, remission induction.

Supplementary Figure S2 Event-free survival of paediatric patients with ALL grouped by diagnostic delay, n=182 (MNH, Tanzania, 2016–2020).


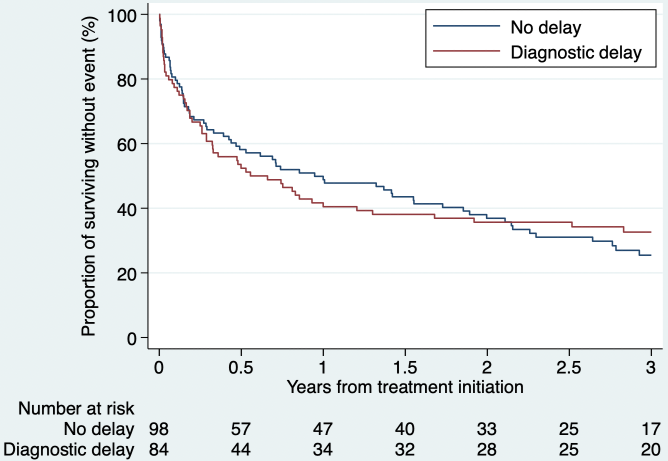


Log-rank test, p=0.87

The Kaplan-Meier curve describes the EFS of paediatric patients who initiated treatment for ALL at MNH from January 1, 2016 to December 31, 2020, grouped by diagnostic delay (diagnosis >7 days after MNH admission). This included 182 patients who were diagnosed at MNH. The difference in the EFS was not statistically significant (Log-rank test, p=0.87).

Abbreviations: ALL, acute lymphoblastic leukaemia; EFS, event-free survival; MNH, Muhimbili National Hospital.

Supplementary Figure S3 Event-free survival of paediatric patients with ALL, n=202 (MNH, Tanzania, 2016–2020).


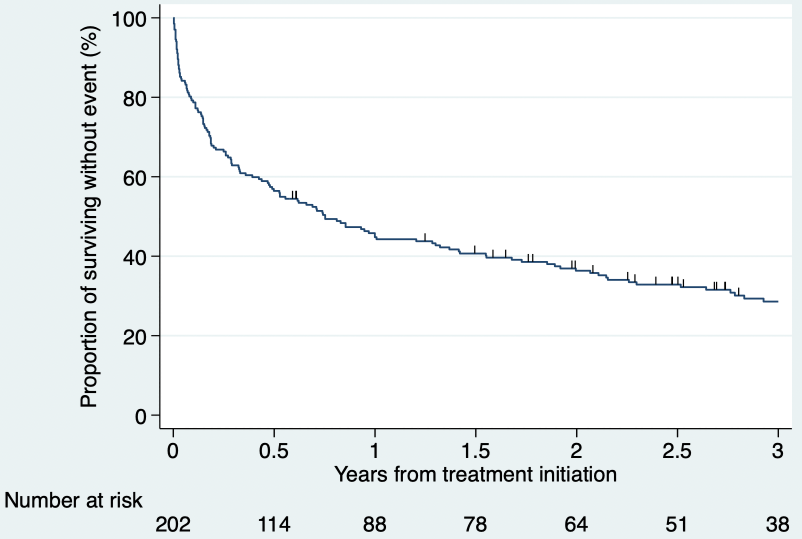


The Kaplan-Meier curve describes the EFS of paediatric patients with ALL who initiated treatment at MNH from January 1, 2016 to December 31, 2020. The events were defined as death, relapse, being sent home for palliation, or abandonment of treatment. The median follow-up period was 41 months. The median EFS was 275 days (95% CI: 193–517), and the 2-year EFS was 36.3% (95% CI: 29.7–43.0). The vertical bars indicate censored observations.

Abbreviations: ALL, acute lymphoblastic leukaemia; EFS, event-free survival; MNH, Muhimbili National Hospital.

Supplementary Figure S4 Event-free survival of paediatric patients with ALL grouped by sex, n=202 (MNH, Tanzania, 2016–2020).


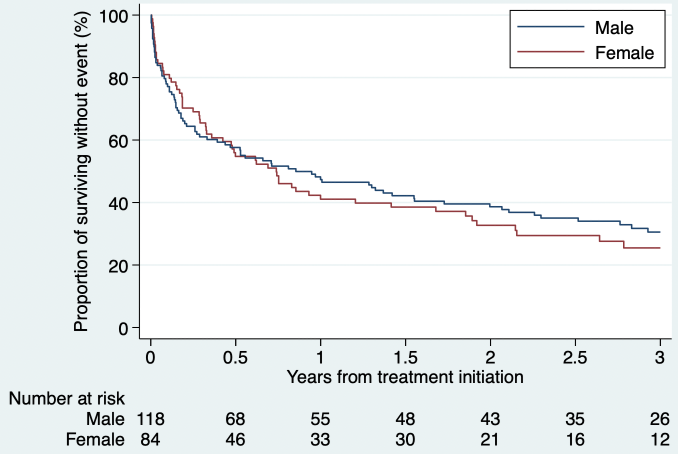


Log-rank test, p=0.48

The Kaplan-Meier curve describes the EFS of paediatric patients with ALL who initiated treatment at MNH from January 1, 2016 to December 31, 2020, grouped by sex. Although the difference in EFS was not statistically significant, male patients had higher EFS than female patients (Log-rank test, p=0.48).

Abbreviations: ALL, acute lymphoblastic leukaemia; EFS, event-free survival; MNH, Muhimbili National Hospital.

Supplementary Figure S5 Event-free survival of paediatric patients with ALL grouped by where patients started treatment (n=255) (MNH, Tanzania, 2016–2020).


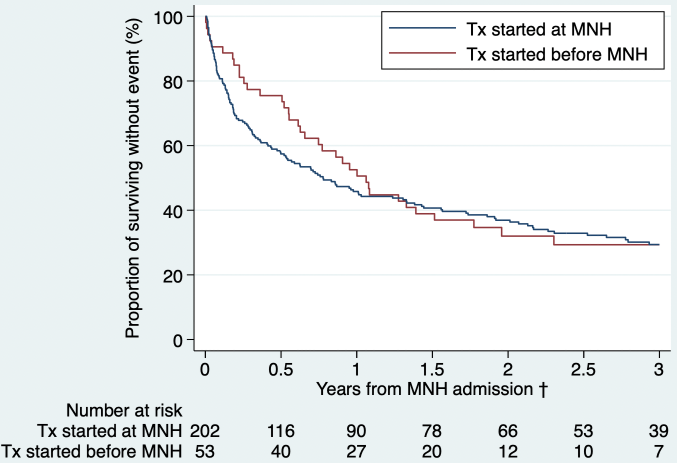


Log-rank test, p=0.56

† The date of data entry for these survival curves is the date of MNH admission, and not the date of treatment initiation at MNH.

The Kaplan-Meier curve describes the EFS of paediatric patients, grouped by where patients started treatment. This included 202 patients who started treatment at MNH, and 53 patients who had started ALL treatment at previous hospitals. Those 53 patients consisted of 6 patients who received cytotoxic chemotherapy and 47 patients who received only steroids. The difference in the EFS was not statistically significant (Log-rank test, p=0.56).

Abbreviations: ALL, acute lymphoblastic leukaemia; EFS, event-free survival; MNH, Muhimbili National Hospital; Tx, treatment.
